# Co-designing new tools for collecting, analysing and presenting patient experience data in NHS services: working in partnership with patients and carers

**DOI:** 10.1186/s40900-021-00329-3

**Published:** 2021-11-27

**Authors:** Nicola Small, Bie Nio Ong, Annmarie Lewis, Dawn Allen, Nigel Bagshaw, Papreen Nahar, Caroline Sanders, the DEPEND team, Damian Hodgson, Azad Dehghan, Charlotte Sharp, Will Dixon, Shôn Lewis, Evan Kontopantelis, Gavin Daker-White, Peter Bower, Linda Davies, Humayun Kayesh, Rebecca Spencer, Aneela McAvoy, Ruth Boaden, Karina Lovell, John Ainsworth, Magdalena Nowakowska, Andrew Shepherd, Patrick Cahoon, Richard Hopkins, Goran Nenadic

**Affiliations:** 1 NIHR School for Primary Care Research, University of Manchester, Manchester, UK; 2 Patient and Public Involvement and Engagement Contributor, Manchester, UK; 3 Department of Global Health and Infection, Brighton and Sussex Medical School, University of Sussex, Brighton, UK

**Keywords:** Patient and public involvement and engagement, Patient participatory groups, Patient experience, Co-design, Co-production, Digital participation, Qualitative health service research, Mental health services, Rheumatology, Primary care

## Abstract

**Background:**

The way we collect and use patient experience data is vital to optimise the quality and safety of health services. Yet, some patients and carers do not give feedback because of the limited ways data is collected, analysed and presented. In this study, we worked together with researchers, staff, patient and carer participants, and patient and public involvement and engagement (PPIE) contributors, to co-design new tools for the collection and use of patient experience data in multiple health settings. This paper outlines how the range of PPIE and research activities enabled the co-design of new tools to collect patient experience data.

**Methods:**

Eight public contributors represented a range of relevant patient and carer experiences in specialist services with varied levels of PPIE experience, and eleven members of Patient and Participation Groups (PPGs) from two general practices formed our PPIE group at the start of the study. Slide sets were used to trigger co-design discussions with staff, patient and carer research participants, and PPIE contributors. Feedback from PPIE contributors alongside verbatim quotes from staff, patient and carer research participants is presented in relation to the themes from the research data.

**Results:**

PPIE insights from four themes: capturing experience data; adopting digital or non-digital tools; ensuring privacy and confidentiality; and co-design of a suite of new tools with guidance, informed joint decisions on the shaping of the tools and how these were implemented. Our PPIE contributors took different roles during co-design and testing of the new tools, which supported co-production of the study.

**Conclusions:**

Our experiences of developing multiple components of PPIE work for this complex study demonstrates the importance of tailoring PPIE to suit different settings, and to maximise individual strengths and capacity. Our study shows the value of bringing diverse experiences together, putting patients and carers at the heart of improving NHS services, and a shared approach to managing involvement in co-design, with the effects shown through the research process, outcomes and the partnership. We reflect on how we worked together to create a supportive environment when unforeseen challenges emerged (such as, sudden bereavement).

**Supplementary Information:**

The online version contains supplementary material available at 10.1186/s40900-021-00329-3.

## Plain English summary

The way we collect and use patient experience data is important because of concern that patients and carers may be excluded by the limited ways it is currently done in NHS services. In this study, we worked in partnership with researchers, staff, patient and carer participants, and patient and public involvement and engagement contributors, to co-design new tools for the collection, analysis and presentation of patient experience data. We focused on services for people with musculoskeletal conditions and services for people with severe mental health conditions. Our PPIE group, formed at the start of the study, represented a range of relevant and diverse health experiences from patients and carers of specialist services, and primary care. The aim of this paper is to share our experiences from working in partnership with our PPIE contributors on the co-design work of the study. Illustrations of how the PPIE activities added crucial insights in the shaping of the tools are given alongside the research data from patients, carers and staff participants. We experienced some challenges during the project. We discuss how we managed to work together to create a supportive environment when unforeseen challenges emerged (such as, sudden bereavement). Our experiences of developing multiple components of PPIE work for this study demonstrates the importance of tailoring PPIE to suit different settings, and to complement people’s strengths and capacity. It also shows the value of bringing diverse experiences together enabling a shared approach to co-design. Researchers and PPIE contributors wrote this paper jointly.

**Supplementary Information:**

The online version contains supplementary material available at 10.1186/s40900-021-00329-3.

## Background

National policy encourages the embedding of Patient and Public Involvement (PPI) in research as a means to improve both the relevance and meaningfulness of applied health services research in England [1], and to ensure research improves the nation’s health and wellbeing [2, 3]. Public involvement in research has previously been defined as ‘research being carried out **with** or **by** members of the public’, including patients and carers, ‘rather than **to**, **about**, or **for**’ them (Pg. 1 [4]). Patient involvement is commonly enabled through establishing a dedicated group to provide inputs for the study duration with public contributors sometimes included as co-investigators to inform the study design from the earliest stage [5, 6]. However, concerns remain that PPI is exclusionary, challenging the relevance and meaningfulness of such work [7]. The National Institute for Health Research (NIHR) have produced a ‘Values and Principles’ of framework of evidence-based practice for PPI [8], and the publication of UK Standards for Public Involvement [9], has been designed to help to address recurring challenges to enable more inclusive and collaborative working, as well as supporting best practice [10].

A number of studies have assessed how patients and carers can be meaningfully involved as contributors to research from pre-funding, through to the end of the study and beyond [11–13], demonstrating the value of PPI and engagement (PPIE) input for shaping research, co-developing (that is, jointly developed), and delivering impactful outcomes [13, 14]. The number of PPIE Journals are increasing [15], and the inclusion of the GRIPP2 reporting checklist and other tools have improved reporting, yet more needs to be done regarding impact assessment [16–19]. Co-production is one approach of obtaining multiple perspectives and sharing views, reflection, learning, from the start to the end of the research [20–22]. This approach is rooted in participatory research, enabling people to become involved in the shaping, design and testing of new healthcare interventions that are patient-centred [23]. Co-design seems to be most effective as a method, when the importance of the relational aspect to involvement are emphasised [24]. Co-design involves close collaboration between PPIE contributors, and the research team [25, 26]. This happens through: sharing of power, perspectives and skills; respecting and valuing everyone’s knowledge; and reciprocating, building and sustaining relationships [20, 22, 27]. Further features are essential: ongoing dialogue; joint ownership of key decisions; relationship building; valuing and evaluating [20, 28], and working with PPIE contributors to encourage a reflective culture. When designing digital health interventions, these should equally be based on patient and carer experiences [29–31]. Finally, PPIE in co-design is context dependent [32, 33] and thus it is important to outline the approach taken in the co-design of an intervention.

### Context of the involvement in co-design approach in the research study

In this paper, we focus on the PPIE and co-design components of a project entitled ‘**D**eveloping and **E**nhancing the Usefulness of **P**atient **E**xperience and **N**arrative **D**ata’ (the **DEPEND** study: [34]). While a dedicated PPIE group was active throughout the mixed-methods study, it was particularly important in the co-design of the new tools and associated guidance (the ‘toolkit’) and their evaluation, to ensure that the perspectives of patients and carers were at the heart of the study. This paper outlines in detail the significant PPIE component in the co-design approach in relation to three aspects: the process; the research components (i.e. the tools co-designed for collecting, analysing and presenting patient experience data); and the impact on the research partners, that is the research team and the PPIE contributors as advisers to the study.

The DEPEND study was developed in response to a dedicated call from the National Institute of Health Research whose brief posed the question: “What research is needed to make patient feedback data more credible and useful?” [35]. Patient experience data is currently routinely collected within the NHS using a range of methods, including the national Friends and Family Test (FFT) [36, 37], the Picker survey [38], and many condition-specific [39], or organisation-specific online feedback surveys [40], some of which are validated. While the attention paid to patient experiences of health care is welcome, a number of issues remain. Research has shown that health care staff are often sceptical of the relevance of patient experience data to local services [41]. Moreover, it is argued that the commonly used formats used to collect experience data tend to exclude patients and carers who might not be able to report their experiences of care.

The evidence suggests that patient experience should be measured in terms of the journey as experienced by the patient in order to capture transitions in care and continuity [42, 43]. This understanding of the dynamics of long-term health conditions, be they mental and/or physical, demands a re-think of what constitutes meaningful feedback [42]. Similarly, recent research on the measurement and improvement of patient experience in primary care [44] and specialist services [45] highlights that surveys may be insufficient to fully capture patient feedback. It has also been suggested that surveys should be made more useful and relevant to staff, and alternative feedback methods should be developed to suit context and staff roles [35, 37, 46]. Increasingly, NHS organisations collect large amounts of data, yet the analysis and reporting of patient feedback is variable because of inefficiencies, lack of expertise, capacity and resources [34]. This is particularly the case with narrative data and the extensive store of patient stories now available has prompted some studies to develop ways of synthesising this material so that it can be used for service improvement. Exploring alternative methods, such as adopting a machine learning classification approach has been reported in the literature [47–49]. The DEPEND study builds on the aforementioned literature and this is reported elsewhere [34] but in essence it used a collaborative approach to co-design by inviting staff, patient and carer research participants, and PPIE contributors, to share experiences, knowledge and resources, to co-design new tools for collecting, analysing and presenting patient experience data in NHS settings.

**Fig. 1:**
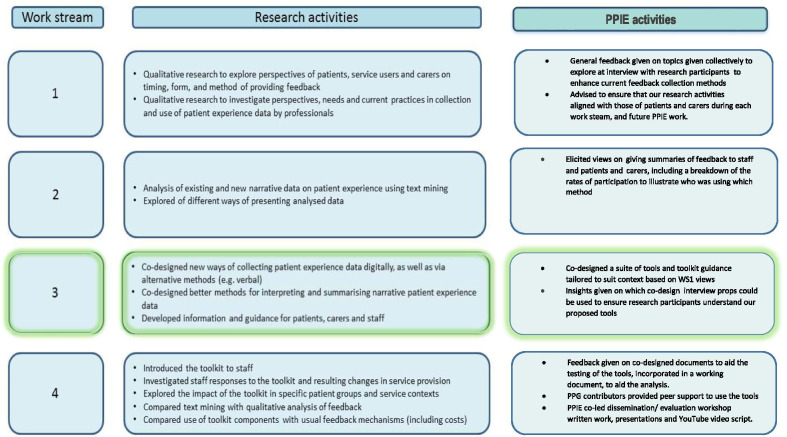
Overview of work streams for the research activities and PPIE activities: the co-design work is shown as work stream 3

The aim of the involvement component to the DEPEND study was to inform the research process within each of the four parts (‘Work streams’) to the study to ensure integration of involvement into the intervention development process from the start (see Fig. 1). This paper will specifically report on the PPIE involvement in the co-design work stream 3 of the study to show how each PPIE co-design activity informed the shaping of the new tools, theoutput and the impact. We draw on exemplar data extracts from the staff, patient and carer research participants collected through focus groups and interviews, to illustrate how our PPIE group, informed the subsequent co-design of the intervention. Further, we also report how our PPIE contributors chose specific roles based on their expertise and capacity that impacted on their levels of involvement in co-design. Lastly, we reflect on our experiences as a partnership.

## Methods

This paper focuses on the co-design element of the DEPEND study and has been guided by the GRIPP2 reporting guidance to improve the reporting of PPIE and transparency [16]. The GRIPP2 checklist (long format) was used as a guidance of reporting PPIE in this paper (See Additional file 1). Our paper is structured thematically illuminating how involvement derived insights informed the co-design process, analysis and outputs. It was jointly written with the researchers and three PPIE contributors (AML, DA, NB) to offer a shared reflection on the PPIE in co-design process, the research outputs, and the significant impact on the research partnership.

### Research context, co-design approach and research participants

Full NHS Research Ethics Approval was obtained for the DEPEND study (Black Country NRES committee in West Midlands ref: 16/WM/0243), and all participants gave written consent.

The context for the DEPEND study is represented by four sites: Site A: a large Acute Trust (focusing on rheumatology outpatients); Site B: a smaller Mental Health Trust (focusing on a community mental health team (CMHT), and an outpatient clinic (OPT)); Site C1 and C2: two general practices within the catchment area of Site A and Site B. This aligns with the NHS Outcomes Framework [50], which highlights key improvement areas for ensuring people have positive experiences of care in CMH services, OPT clinics, and GP services.

The co-design approach adopted draws conceptually on Experience-Based Design (EBD: [51]) theory that defines experience as a reconstruction, or a reconstitution of something that individuals have lived through. Meaning is given to that experience in a reflective and retrospective manner through words. This specific kind of knowledge is gained from close and direct personal observation, or contact, in order to design improved services and a better experience for patients or carers. It is conceived as a partnership, involving researcher, staff, patient and carer research participants and PPIE contributors, working as co-designers of services throughout the change process [51].

Patient research participants were recruited through staff in the clinical sites; staff research participants were recuited via clinical research leads at each site. Research participants were invited to take part in either a focus group or a face-to-face individual interview, or both, as determined by participant preference. Qualitative data collected from staff, patients and carers participants from the first phase of data collection was summarised and discussed in follow-up co-design focus groups, with 57% of staff and patient and carer participants having previously taken part in the qualitative components in the first phase (see Table 1). We achieved a diverse sample of staff participants with roles in management and patient experience, clinical and information technology. The total number of people who participated in the three data collection phases for DEPEND is reported within the final report [34].

**Table 1:** Participants by sites: co-design of new tools for collecting, analysing and presenting patient experience data

Participants	Sites				
	A	B	C1	C2	Total
Staff focus groups	10 (5)	12 (5)	9 (6)	14 (7)	45 (23)
Total staff	10 (5)	12 (5)	9 (6)	14 (7)	45 (23)
Patient focus groups	0	12 (7)			12 (7)
Patient interviews	8 (7)	0			8 (7)
Total patients	8 (7)	12 (7)			20 (14)
Grand total	18	24	9	14	65 (37)

Number of participants that had taken part previously is in brackets; shaded area indicates data from patients in sites C1 and C2 were not collected for the co-design phase

### Establishing a PPIE group

At the start of the DEPEND study, a PPIE group was created with three PPIE contributors who had experience of specialist services for musculoskeletal health conditions, and five PPIE contributors receiving treatment for serious mental health conditions; two of five PPIE contributors represented a dual perspective as carers and patients in the study. Two PPIE contributors were co-applicants to the study (AML and DA); they attended and contributed to the bi-monthly project management meetings, and the quarterly Study Steering Group, and favoured a more active role in the group. Both contributors were experienced in PPIE work having previously partnered with research teams to co-design digital interventions and disseminating the research findings on behalf of the research team [52, 53].

Representatives from two Patient Participatory Groups (PPGs) were next recruited as PPIE contributors to the study via the two primary care sites Lead clinicians (Site C1 and Site C2: [54]). The PPG in Site C1 was a relatively small group with nine members, four of whom chose to support the DEPEND study as involvement contributors. Conversely, the PPG in primary care site C2 comprised of 40 + members: 7 members who regularly attended face-to-face PPG meetings chose to support the DEPEND study [55]. The Assistant Practice Manager managed the PPG, circulating the minutes produced by the PPG coordinator (and co-author of this paper, NB) and liaised with members.

All PPIE contributors to the study were reimbursed for their time (INVOLVE rates: [56]) and travel. A PPIE document was developed and modified as the study progressed in line with an established Terms of Reference document developed by the Primary Care Research in Manchester Engagement Resource (PRIMER: an established PPIE group funded by the NIHR School for Primary Care Research at the University of Manchester [57]). The document adhered to the principles of roles and responsibilities of INVOLVE [8], and local Faculty guidance [58].

Face-to-face involvement meetings were held in groups for each phase of the research study at the University or at each primary care site so PPIE insights could feed directly into the four work streams (see Fig. 1). Altogether, four PPIE group meetings were held at the University, and five PPG meetings at each site. Each PPIE/ PPG meeting was devoted to a specific topic that reflected the objective of each work streame in the DEPEND study [34]: (1) collation of current perspectives of collection, analysis and presentation of patient experience data; (2) co-design of the toolkit components and accompanying bespoke guidance; (3) testing out and evaluation of the new tools; and (4) PPIE-led dissemination activities. Research documents and an agenda were provided in advance of the meeting by email, or mail, and hard copies were available on the day. Each PPIE activity and contributions were recorded by the PPIE Co-ordinator and project researcher (NS), and circulated to everyone attending the meeting to check for accuracy. Insights from previous discussions were included in the prepared slide set to show how these informed the ongoing research data collection and qualitative analysis. Following the meetings, a working document was circulated where PPIE preferred roles and actions were outlined, and lessons learnt recorded. NS facilitated regular communication with the group via email, and ensured core documents were available for comment via Drop-Box for Business, email, telephone, or face-to-face discussion, enabling a good relationship with the researcher to be sustained.

### Steps in the PPIE in co-design process

During the co-design phase, two PPIE meetings, and two PPG meetings were held at each site. From the beginning, a modified EBD approach [51] created the right environment and ethos for the meetings to take place with our PPIE contributors as we adhered to the collective shared learning principles of co-design. The relationship that developed helped this to run smoothly. The PI, CS, chaired each meeting, with support from two members of the research team, NS and PN. Ahead of the meetings, we developed summaries of PPIE insights from phase 1 data collection with staff, patient and carer research participants, and presented these findings to the PPIE group using PowerPoint, to trigger co-design discussions. We tailored each co-design slide-set to reflect views from our PPIE partners and staff, patient and carer research participants from each of the sites. An example is shown in Additional file 2. We found that by including a slide on ‘what we said, what you said’, helped to trigger discussions ahead of showing the research data collected from each of the sites.

Of note, co-design data was not collected for patients and carers using the services of Sites C1 and C2 as these were not collected in work stream 1. These perspectives were captured via Site A and B as these patients and carers used the services of Site A and Site B. The PPGs participation in the trigger discussions ensured that Site C1 and C2 could give their perspectives and interpretations. This influenced our co-design analysis.

Members of the research team, NS, PN and CS, and PPIE/ PPG contributors, individually and collectively shared thoughts about what might work in practice, challenged ideas and raised concerns together in ways that led to change (see Table 2 for an overview of these recommendations). Preferred roles in the co-design process and output were negotiated amongst the group members during each meeting; these roles evolved as the PPIE component progressed through the co-design to testing work stream. Some of the more active roles we outline in the reflective narrative to the paper.

The final step involved a written summary of each co-design discussion prepared by NS and emailed to the PPIE contributors, and wider research team for review.

### Co-design analysis

All qualitative research data were transcribed, collated and analysed thematically drawing on a grounded theory approach [59], and using NVivo11 qualitative analysis software. Thematic coding was conducted by the researchers (NS, PN, CS, BNO) producing distinct accounts for each research participant group (patients, carers and staff) in each site. The co-design analysis on the research data and the PPIE insights was conducted in parallel to the research activity for work stream 1 and 2 (see Fig. 1), to enable issues and preliminary themes to be explored with participants and our PPIE contributors. We used trigger discussion slides showing the results of the research data collected from work stream 1 and the PPIE insights (from previous meetings) together with preliminary themes from the analysis; these insights were then incorporated in the specific co-design analysis following each meeting. The impact of our approach to the analysis was that the PPIE contributors experienced first-hand how their insights fed in to the co-design of the tools and the subsequent testing of these in multiple NHS sites. Detailed notes written following each co-design PPIE meeting were imported to each NVivo unit to support the main thematic analysis. Some more experienced PPIE contributors chose to email reflective thoughts on the minutes of each involvement meeting and these were also incorporated in the analysis. Preliminary themes from the analysis from work stream 1 were used as a basis for discussion at the co-design qualitative interviews and focus groups, and meetings with PPIE contributors, where links and distinctions across the multiple groups of participants and sites were explored (see Additional file 2). We now turn to the key themes that emerged from the co-design analysis.

## Results

### Capturing experience data

As the FFT is a nationally mandated patient feedback tool [36, 37], we sought specific feedback from our PPIE group on how to enhance the FFT question digitally by changing the response scale to capture emotional feedback collected via emoticons, and adapting the FFT free text question to invite more meaningful comments.

#### Adapting the FFT response scale to capture emotional feedback

PPIE contributors liked that the proposed FFT question and how response could be enhanced to capture emotional feedback to encourage more people to use it*.* The issue of how best to capture people’s feelings about the healthcare they received was tackled differently by PPIE contributors and research participants. In general, the PPG contributors in both primary care sites considered the use of emoticons appropriate, and feasible, as their practice was currently collecting feedback digitally in this way. Further, they also recommended that four instead of five options on the FFT response scale should be offered ‘*as people tended to go for the middle ranking where 4 makes the respondent go one way or the other*’ (researcher note). Likewise, the PPIE SMI contributors described making the FFT question and response via the interface simple, quick and friendly to use as ‘*some people like to use simple emoticons for feedback with an option for adding brief text’ (researcher note).* Some PPIE MSK contributors described preferring an alternative visual system, for example, traffic lights to express emotional feedback as it was felt that patients with MSK might experience discomfort to touch the iPad screen and that emoticons might not represent their pain*.* These views on appropriateness of using emoticons to collect emotional feedback resonated with the majority of staff participants working in mental health services (site B) where they talked about specific sensitivities of collecting feedback digitally in their context:

‘I don’t know whether necessarily in mental health they’re always the best tool for measuring satisfaction, you know, for somebody who is suffering with an episode of depression, there’s going to be nothing that makes them smile from ear to ear, so to see it as an emoticon to measure the most satisfied with a big smiley face, it’s not something that’s right at that time’ (Site B CMHT, ID349, Staff FG).

As a result of these initial discussions, different options were explored by PPIE contributors, and in the subsequent co-design interviews with staff and patients. This led to a joint decision to test out emoticons with traffic light colours on the interface in four settings (Sites A, B OPT, C1 and C2: see Additional file 3], but not in the mental health setting, as these were viewed as inappropriate.

#### Enhancing the FFT free text question to elicit meaningful feedback

All PPIE contributors and research participants voiced how it would be most useful for a new digital tool to capture both negative and positive comments within the FFT free-text box. This emerged from the PPIE suggestion of using a gratitude journal form of feedback, where the option for one piece of positive feedback and one piece of negative feedback is given:

‘The negative feedback is clearly what you want to know where things could be improved. The positive feedback is where you don’t want to make unnecessary changes when things are working well, but you need to identify those areas where the patient thinks things are working’ (Site A, ID107, patient).

Our PPIE contributors also felt that capturing both positive and negative feedback from different service elements within each setting would be useful to examine:

‘It was thought a good idea to capture the positive and negative feedback from different areas of the service e.g. experience at reception, experience with doctor etc. to explore the usefulness of feedback data.…’ (PPG Site C2, researcher note).

As a result of these discussions the FFT free-text question was enhanced to capture positive and negative free-text comments via a digital interface to replicate the check-in process in place in three sites (B OPT, Sites C1 & C2: see Additional file 3).

### Enhancing digital and non-digital tools

All PPIE contributors and research participants could give views on the use of feedback via the following tools: iPad with self-standing kiosk; text message; dedicated phone line; face-to-face discussion; pen and paper; and the use of the site-specific website. This suite of FFT tools was based on the phase 1 data collected. The possibility of building in a feedback period was initially suggested by our PPIE SMI contributors, which would allow patients and carers to provide comments pre and post consultation in each NHS setting:

‘Waiting time could be used for feedback. Pre and post feedback may be based on previous experience, or lack of experience, or expectations for the appointment. Subsequent visits can include the after-service experience’ (researcher note).

This view was shared with our PPIE MSK contributors so that people could be invited to give feedback in their own time:

‘There was group agreement that people should be invited to give feedback pre, during and post appointment and in their own time… Some contributors described people on medication with side effects that might make them confused… Pictures alongside each instruction might be of help’ (researcher note).

In the PPG discussions, a few individuals asked if the digital Patient Access System that now is widely adopted in primary care could house our FFT feedback survey. Further alternatives were a phone app, providing a URL from the site website, or text message following a consultation. Some contributors liked the idea of enabling SMS text messages for collecting feedback:

‘One PPG member said they get sent text messages via [site A] asking for patient feedback but find them very repetitive. The group thought flyers to be useful props to remind people to feedback via different methods, but ‘texts are a nightmare’’ (PPG site C2, researcher note).

This suggested that some guidelines of how and when to send these messages should be developed if we were to test this tool. While enthusiasm for digital methods was high, PPIE contributors and research participants felt that non-digital tools should be offered in parallel. Physically situating different tools alongside each other was seen as another possibility to give people choice of feedback method:

‘But I guess if you've got a [digital] stand like that, I mean I don't know, I'll show it to you when we go down, when you leave, because I was looking at that stand there, there's nowhere to put pen and paper, however, the one downstairs it's got like a little thing on it where you could actually put…you can have your screen but you could actually have pen and paper next to it’ (Site A, ID354, Staff FG).

Many PPIE contributors and research participants felt that this dual approach would enhance the level of participation as patients could choose their preferred method and that the feedback tools offered in each site should be ‘context sensitive’. Thus, the differences between health conditions and their impact, therapeutic relationships and care settings should be taken into account*.* Integrating feedback within the therapeutic relationship in Site B was seen as another option to respect the person in a holistic way:

‘I have a CPN [community psychiatric nurse] nurse and she comes to see me every 2–3 weeks about my tablets and talks to me... then you can discuss things, can’t you?’ (ID208, Site B, patient).

This demonstrates that trust and understanding of a patient’s life facilitate gaining feedback on the delivery of care, and the design of new tools should reflect this. Consequently, a new process for eliciting feedback within CMH services was co-designed to encourage the recording of verbal feedback from patients and carers that may have been excluded using current methods. In brief, each care coordinator would ask a patient a couple of trigger questions at the end of their home visit in order to allow comments on people’s experience of the service. The care co-ordinator would then give a brief explanation to say that the team wants to improve information they collect about people’s experiences of services. Responses to the trigger questions were to be recorded in a dedicated field within the electronic care record used by the CMHT (see Additional file 4).

### Ensuring privacy and confidentiality

For PPIE contributors, privacy and confidentiality were considered equally important to encouraging patient feedback via the new tools. The key issue was the location of the new digital kiosk. Many contributors expressed it should be located in a private cubicle in appropriate reception or clinical areas, clearly signposted and inviting:

‘PPGs liked the idea of using a digital screen for giving feedback as they use screens to check-in for appointments. Another contributor also liked the idea of having a private cubicle, would this be possible? Like a passport photograph cubicle, to enter and give feedback. There was collective agreement that privacy and confidentiality are equally important to encourage people to feedback to services’ (PPG Site C2, researcher note).

As a result of these insights and preferences, joint decisions were made on testing the new tools and they were placed in areas of privacy. They were monitored by the observation measures in place during the testing phase.

### Co-design of a suite of new tools

The majority of PPIE contributors and some patient and carer participants talked about using guidance, information and signposting to motivate patients and carers to give feedback using the new tools:

‘The importance of presenting “feedback about the feedback” was met with group laughter but agreed to be a crucial element to the guidance produced to accompany the new tools’ (PPI MSK, researcher note).

It was also voiced that any guidance could work to encourage not only more but also better quality feedback. This idea was adopted in the form of a colourful poster in four sites and later a larger poster to support use of the new tools in two sites (C1, C2: see Additional file 3):

‘It was thought that more striking colours and something simple on the poster needed to be used—even something like “Please give us your feedback” with a big arrow pointing to the kiosk’ (PPG Site C1).

The PPGs gave crucial insights on how to present and update the content of the poster ensuring it remained current amidst the plethora of information on display. Discussions about providing this information led to displaying examples of positive comments on a later version of the poster*.* Several PPIE contributors and research participants talked about the need to provide hands-on support, especially for older people or others who might be less confident in using digital devices:

‘I think it just depends on people’s... well, partly the age, isn’t it, and their abilities. I mean, obviously some older people will struggle. I mean, I do intend to get computer literate again, but I’ve not been able to use my computer, and I mean, my skills are quite basic... If there’s like some support available, so for example when you think of the supermarket when you use the... self-serve, and there’s usually someone there, and it comes up on screen and so if there’s someone there if you’re stuck or something flags up’ (Site A, ID115, patient).

PPIE contributors and staff participants explored ways of providing support for patients and carers to use the kiosk. Staff reported that the majority of patients and carers would not use the kiosk unless they were asked to do so. Use increased once the larger colourful laminated poster with guidance notes was placed above the kiosk and a PPG member (PPIE contributor supporting the study) promoting the use of the kiosk was in post:

‘The PPG was active in promoting the kiosk in the reception area. Five people walked up to the poster (this has never happened before with the smaller A4 landscape poster), read it for a few minutes, then used the kiosk without being prompted by either of us’ (Site C2, observation note).

Two PPG members attached to Site C2 provided peer guidance on how to use the kiosk during the busiest clinics. Having this component in place allowed data capture on the acceptability of and continued engagement with digital feedback. We subsequently organised for a volunteer attached to Site B OPT to provide peer support during the testing phase. This role increased rates of ditial kiosk participation in Site C2 [60, 61], and aided implementation in both settings as it responded to staff concerns about the impact of the new tools on their workload:

“... if I’m having to come away from my desk to talk them through it, even through the glass, that is going to take me away from the phones, it’s going to take me away from what I’m supposed to be doing” (Site B OPT, ID262, FG).

Our recommendation that information and bespoke guidance should be provided ready for the set-up of the new tools was to be monitored and adapted to incorporate the feedback data throughout the testing period [34, 60, 61]. As part of the later phase of the co-design and implementation phase, visual feedback reports that included summaries of quantitative data and free text comments were split by sentiment (positive and negative comments: [34]). In line with PPIE and staff preferences, these monthly reports were tailored to the different service contexts.

A summary of main points of feedback and recommendations made by the PPIE contributors to shape the research components by each theme through co-design is shown in Table 2 (Appendix). These crucial insights also helped us to tailor interview props for the co-design interviews (Additional file 2, Fig. 3).

### Dissemination and evaluation

A special PPIE dissemination and evaluation workshop in February 2018 was held, with presentations on the DEPEND study research findings. Nineteen members of the public attended the workshop, with representation from the study sites and multiple PPIE groups and networks across Greater Manchester. Members of our PPIE group co-presented at the workshop and co-facilitated discussions on our findings from DEPEND. Feedback on our reflections resonated with the public audience:

‘I found that the way those presenting conveyed how much this project has meant to them, the emotions of it, was very moving and inspiring’ (workshop attendee).

We also used this workshop to premier an animated video reporting the study findings [62], and this was received positively:

‘I loved the animation, it is a great visual way to present information in increments, that some people might not read through if it were just text, the humour in it helped to keep it engaging too’ (workshop attendee).

We were able to use the comments and contributions to make final improvements to the animation prior to general release.

Following the PPIE workshop, two of our PPI contributors, AML and DA, co-presented with NS, our experiences of working together on DEPEND at an International PPIE conference [63], and spoke about some of the challenges and successes. The presentation highlighted the importance of joint working between PPIE contributors, researchers and NHS staff in order to co-design new tools that have the best chance of working in practice.

**Fig. 2:**
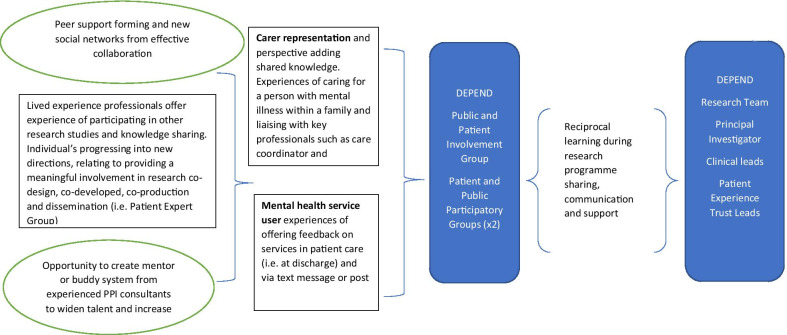
PPIE reflection model by DA

Following the co-design and testing phases of DEPEND, we conducted a meeting with members of our PPIE group to hear collective and individual views on their experiences of the DEPEND study, and in particular the co-design model. Overall, the process and the outcome of PPIE in the DEPEND study was successful: strong and trusting relationships between PPIE partners and researchers had developed over the 2-year study. The PPIE partners made valuable contributions to, and provided important insights into each work package, ensuring that research priorities aligned with those of patients and carers and these enabled recommendations for delivering future PPIE work to be developed. All of our PPIE contributors commented on how much they had enjoyed being part of the team and that the experience had been rewarding. Participating in the DEPEND study had taken various forms for our PPIE contributors and examples are highlighted in a visual representation developed by one of our more experienced PPIE ‘lived experience’ co-investigators, DA, a co-author on this paper (See Fig. 2).

The model contains some personal involvement reflections and outcomes (green oval) and learning points (box) useful for future PPIE in co-design work from the mental health patient and carer perspective. The model emphasises the nuanced aspect to involvement in a study, what it may achieve for some more exprienced contributors and how. It is important to note that DA prefers the term ‘lived experience’ PPIE contributor; others in our PPIE group prefer the sole term ‘contributor’ to PPIE, and the PPGs prefer ‘volunteer’ or ‘member’. These terms were captured in our Glossary. We have jointly written this paper using all preferred terms.

Peer relationships flourished with many PPIE contributors contacting one another in between meetings, and ongoing communication between the PPIE contributors and the research team enabled the partnership to be sustained during the funded 2-year period, and beyond. Other PPIE contributors to DEPEND, realtively new to PPIE, spoke of becoming more confident as they attended more groups at the University as the research progressed.

Specific roles were allocated to those PPIE contributors based on preferred activities. DA helped us to recruit carer participants within Site B using DA’s Patient Expert Group networks. This enabled us to widen our recruitment with DA spending time on site with the researcher at carer events held by Site B and attending meetings with the carer lead at this site. During year 2 of the study, DA spent dedicated time working at the University with NS and contributed to the shaping of toolkit documents (see Additional files 3 and 4). Our PPIE co-applicant, AML, also spent a significant amount of time reviewing components for the toolkit. Other PPIE/ PPG contributors gave crucial practical support throughout the evaluation and dissemination phase to DEPEND. One PPG (Site C2) took a lead peer support role that helped to promote and sustain kiosk use during the evaluation of the tools phase. Of note, they witnessed how patients and carers mostly reacted positively to the new tools (these reactions are reported sepately [60, 61]). They passed their observations on to the researchers via email and in-person; these insights were crucial to making sense of the unfolding analysis.

Two PPIE contributors had a lead role in co-designing the animated film and co-delivered a public dissemination workshop.

## Discussion

The DEPEND study involved staff, patients and carers research participants from multiple sites co-producing the research with a core PPIE component embedded within each of the work streams [34]. We were able to adopt a shared approach to co-design through working in partnership with patients and carers, and research participants, to shape a suite of new tools to test the usefulness and relevance of patient experience data in four NHS settings. This paper reports on the most significant and innovative PPIE involvement, namely the co-design work: the enhancement of digital and non-digital methods to collect, analyse and present patient experience data by modifying the FFT question [36, 37]. The DEPEND study adopted a modified EBD approach and the deliberative discussion of the findings of the earlier research phases and the systematic application of shared interpretations allowed the tools to be critically reviewed, developed and refined by the PPIE contributors and research participants. Consequently, the co-design of a toolkit reflected key insights provided by our PPIE contributors alongside the shared analysis of the research data. The sensitivity to the different health conditions and service contexts ensured the adaptability and relevance of the tools across the four sites. Most importantly, the investment in the relationship between PPIE contributors and the research team helped to maintain mutual trust throughout the study. Our study makes a specific contribution to the evolving landscape of PPIE, co-design, and co-production. The novelty of our work lays with successfully embedding a multi-level PPIE strategy throughout the work streams for the duration of the study and beyond that enabled a partnership to evolve with significant impact on the research process, outcomes and the partnership members.

### Our reflections on the effects on the research process, outcome and working in partnership

DEPEND was a complex multi-site study requiring significant PPIE action planning and input throughout each phase. Our PPIE in co-design experience in DEPEND highlights a number of important issues, which we discuss in turn with reference to the current guidance to practice [22], and related research. Investment in the PPIE partnership with patient and carer contributors is key, from the pre-funding stage of research through to the dissemination, and beyond the study [5, 6, 22, 64, 65]. Our findings show the need to tailor PPIE to preferences and values of research partners, and consideration has to be given to how variation in individual needs affects ongoing participation [11, 12, 14, 66]. Having honest and open conversations and creating a glossary of terms at the start of our co-production journey helped us to tailor varied strands of PPIE work, matching specific roles to individuals and working flexibly to optimise participation in each work stream. Some contributors reviewed and contributed to core documents remotely via skype and dropbox, allowing inclusivity and personalised adaptation. The PPGs in Site C2 favoured a specific peer support role, matched to the way they want to engage in their general practice [67]. Recent co-produced research recommends implementing costed external training, especially with regard to establishing a relationship from the start [68].Our PPIE group was diverse with patients and carers with differing levels of PPIE experience. We found this dynamic worked well in terms of assigning roles and responsibilities [22], and tailoring varied strands of work to specific roles based on preferences enhanced the value of the PPIE in co-design as everyone was treated with equal importance [13, 14, 68, 69]. Establishing a joint understanding and clarity of roles was central to managing expectations, especially when sometimes we could not feasibly meet preferences for involvement. It was was hoped that 2 more active PPIE contributors would be involved in co-facilitating the interviews and focus groups but unfortunately this was not possible due to bureaucratic and budget constraits. All our contributors were well supported with comprehensive plans and actions and we demonstrated reciprocity in our approach of joint working by enabling focused discussions in groups [29, 30, 65]. On reflection, being transparent with our thinking helped us to build on the initial investment in the research partnership by sharing perspectives during the co-produced research [20, 21, 23, 24, 27, 28]. Having a dedicated PPIE coordinator with support from at least two members of the research team enabled facilitation of separate co-design meetings so that insights could feed into the unfolding data collection and analysis [25, 29]. We faced some major challenges and upsetting events during the 2-year study. The sudden passing of two of our PPIE contributors, Jane and Neal, made us reflect on the relationships developed during the course of the research and on how to manage difficult situations. Working closely together as researchers and PPIE contributors entails sharing personal experiences and building long-term relationships that are quite different from those that develop when researchers are carrying out short research projects. Researchers, and PPIE contributors, develop a sense of responsibility towards each other and managing expectations can become harder without the necessary support, tools or guidance in place. When a member of a group passes away suddenly, we found it might be difficult to know what to do to support other members of the group, who may already feel vulnerable. We supported each other individually and as a group, enabling people to express bereavement in their own way. Some of these learning outcomes are shown in DAs reflection model (Fig. 1) drawn from a reflective blog on her experiences of working in partnership on the study, conveying the importance of her role and passion for PPIE:

*‘My latest involvement in DEPEND, designed to improve data collection and usefulness, has given me the opportunity of more meaningful ways of becoming involved in community mental health feedback and review specific parts of the work streams. As a team, we have had to deal with bereavement. Neal’s passing highlighted the importance of my role. It was wonderful working with him and we will miss him. It makes you realise how precious life is and how important it is to make the most of the good days when living with mental health challenges. It also highlighted how valuable I feel my role at the university has become and feel very proud of this! It has also raised the important issue of making sure we get support in our work, here at the university, if we are managing other long term challenges’*

The research team also took time to individually and collectively reflect on what happened, and what could we do to make sure we all felt comfortable to continue with the research. Special peer relationships and friendships developed between PPIE contributors. For example, DA and NS, participated in the annual Community Festival at the University to champion their working partnership to the local community [70].

The research team were offered individual counselling at the University, but we all felt a group intervention, where collectively we could continue to talk openly and honestly about what happened with our PPIE contributors would be more appropriate. The PI, CS, reached out to the clinician co-applicant on the research team, who facilitated a debriefing meeting with the researchers and the PPIE group at the University. The wider social responsibility community at the University also supported us all as a group by offering a platform for a co-delivered presentation on our PPIE experiences and to celebrate our achievements to date. Both these interventions helped us to move forward together illuminating the importance of sharing positive and challenging experiences and reciprocal learning. A posthumous award was conferred on our PPIE contributor in recognition of the exceptional PPIE work completed in the development of digital innovations for people with mental health conditions [71].

The absence of guidance and support to deal with such emotionally upsetting events when working as a partnership led to further research funded by the NIHR to explore ways to address this issue given that PPIE is integral to research both in our local communities and beyond [72, 73].

### Benefits of taking a PPIE in co-design approach: future directions

One of the benefits of co-design that emerged in this study was beginning the involvement and design process from the ‘lived experience’: from the reality of everyday work, rather than designing from theory, to ensure that we put patients and carers’ perspectives at the heart of the research study. Having an extended PPIE group incorporating new and experienced contributors was conducive to co-design. The two PPGs exemplified the experiences and preferences of collecting feedback with long-term service use for our exemplar physical and mental health long-term conditions in primary care. The findings were crucial to capturing what tools might work in practice as they were on site. Published studies have similarly reported on the richness that emerges in the research and partnership from adopting PPIE in co-design [32, 33]. Our work resonates with the reciprocal learning reported emphasising the importance of building the foundations for relationships, fostering trust, respect, creating a safe space, to talk about the research and the effects on the partnership [74].

Overall, both the process and the outcome of PPIE in the DEPEND study was seen to be successful by PPIE contributors and the research team, as indicated by the strong relationships that had been forged over the 2-year study. Our PPIE collaborators made valuable contributions to, and provided insights into, each work package, enabling us to develop recommendations for delivering future PPIE work. Issues to be considered in future PPIE activities described by the group included: retention of PPIE contributors; diversity; inclusion; choice of language; and measuring fear, or the stigma of becoming involved in PPIE activities [18, 19, 75–77].

We recommend linking to external organisations who embed PPIE work at the heart of research, such as the NIHR School for Primary Care Research [3], and the National Co-ordinating Centre for Public Engagement [78], where guidance is kept current, to encourage a culture of fair, equitable and meaningful involvement. We further suggest researchers and PPIE contributors make use of the many established online PPIE toolkits, which have been co-produced with patients and carers [21, 24, 58]. Finally, the DEPEND study was conduced before the COVID-19 pandemic, however, the lessons learnt remain as challenges for health researchers worldwide [79]. It is imperative that we continue to share learning with the research community to continue to tackle challenges and to celebrate achievements when working with patients and carers in partnership [80].

We focused on disseminating our PPIE learning throughout the study period through various avenues connected to the partnership, including: the national NIHR Patient Experience Learning Set [35]; a focused PPIE International conference [63], as well as specific events held promoting the use of feedback held at all sites, and at the University. It would have been impactful to hear more from the audience connected to Site B than was possible during the study about our novel tool of asking a trigger question to capture verbal feedback. We struggled to recruit carer research participants, despite having the assistance of a PPIE experienced contributor, DA, with access to many carer third sector organisations. Future research should move beyond recruiting via the organisations networks, which host the research, and invest in knowledge brokering activity with all stakeholders [81, 82].

## Conclusions

We have shown how PPIE in co-design shaped the research process and its materials by outlining specific ways in which it added value to the design and delivery of the toolkit. Our experiences of developing multiple components of PPIE work for this complex study across various health services, demonstrates the importance of tailoring PPIE to suit different settings, and individual strengths and capacity. The contribution of PPGs to PPIE in health services research work is under reported and remains to be explored further. Our study of PPIE in co-design and co-production shows the value of bringing diverse experiences together, and adopting continuous feedback loops that transparently show the way in which PPIE inputs shape the research. Adopting a shared approach to managing challenging situations is suggested as we encountered the need to develop psychological and practical solutions to partnership working with patients and carers. Our approach to PPIE in co-design was wide-ranging and iterative, and this paper includes important personal reflections on the emotional consequences of investing in PPIE for both PPIE contributors and the research team that need to be acknowledged for the process and outcome of meaningful PPIE to evolve and be impactful.

### Supplementary Information

**Additional file 1**. GRIPP2 long form.

**Additional file 2**. Example trigger discussion slides used in the co-design process.

**Additional file 3**. The DEPEND study toolkit.

**Additional file 4**. A new approach to collecting verbal feedback.

## Data Availability

The data generated during or analysed during the current study are not publicly available due to ethical restrictions. All data queries and requests should be submitted to the corresponding author, Dr Nicola Small, SPCR Fellow, for consideration.

## Appendix

See Table 2.

**Table 2:** Summary of theme, feedback and recommendations

Theme	Feedback	Recommendation
Capturing experience data	PPIE contributors liked the fact the proposed FFT question is short and focused PPG (site C2) thought inserting text to indicate the length of survey to encourage people to complete it might be useful PPIE contributors liked the use of traffic light colours to capture emotional feedback via the response scale. Some contributors with MSK preferred an alternative visual system (e.g. traffic lights to signal warnings) to emoticons, as it was explained how patients experience pain to touch screen	Researchers should keep the FFT questions short by only enhancing the free text question Researchers should should show the start and finish of the survey by inserting buttons at the bottom of the screen on the digital interface Researchers might keep the traffic light colours with emoticons via the digital interface, to observe people’s reactions to the enhancement
	PPIE contributors felt being able to record one good experience and one bad experience was a new idea; it was agreed that people would need prompting somehow to do this	Researchers should place text to prompt positive and negative feedback in the free text FFT question and add examples in brackets to help elicit critical feedback by sentiment
Enhancing digital or non-digital tools	PPIE contributors liked the idea of typing on an iPad touch screen with self-standing kiosk but thought this might not be a suitable feedback method on its own, a suite of tools would enable a preferred way of providing experience data PPIE contributors thought text messages might be useful to test alongside kiosk method as a quick feedback tool; PPI MSK contributors warned timings of these text messages would be crucial to avoid unsettling people PPIE contributors felt that the URL to a site-specific website might be useful if linked to appointment feedback, maybe via a text message PPIE contributors with SMI felt having a face-to-face discussion to a trusted person might be a preferred alternative to feedback verbally for those patients and carers who had concerns over using other feedback methods PPIE contributors described the importance of having pen and paper available as an essential non-digital tool	Researchers should offer a suite of tools to cater for preferences, to encourage those patients and carers who might not have previously given feedback using paper to use the new tools: digital self-standing kiosk via iPad touchscreen (sites a, b OPT, c1–2) text message (site c2, site a, c1 texts were disabled during study period) URL to a site-specific website (sites a, c1–2) dedicated phone line* (site b CMHT) face-to-face discussion via their care coordinator (site b CMHT) pen and paper leaflet, or a postcard (sites a–c)
Ensuring privacy and confidentiality	PPIE contributors reached consensus that the location of the new kiosk was important to get right to encourage participation, this needed to be a relatively private location, a separate room or cubicle would be best	Researchers should consider implementing: staff and volunteer roles, feedback time, environment, privacy concerns, especially with the kiosk and iPad, and home visits were all equally considered when deciding the tools to test out and where to place these to ensure privacy and confidentiality, as far as possible: digital self-standing kiosk via iPad touchscreen placed in reception area of GP practice and outpatient clinics (sites a, b outpatients only, c1-2)
		text message (site c2; site a, c1 texts were disabled during study period) enabled via Practice Manager
		URL to site-specific website (sites a, c1-2) sent via text message and placed in PPG newsletter, toolkit flyer and website dedicated phone line, safeguard issues were raised by site b CMHT staff* face-to-face discussion with care coordinator during home visits, to capture experience data using an iPad, and documented in a dedicated field of current system (site b CMHT)
Co-design of a suite of tools with guidance	PPIE contributors thought a colourful poster would be the most effective way to advertise the use of the new tools in all sites PPG C2 gave examples of positive and negative experience data to include on the first iteration of the poster on the premise data would be replaced with real-time data captured by the new tools Contributors advised that the poster and other guidance to advertise the new tools would need to be near the kiosk; PPG C1 contributor suggested we might use an arrow pointing to the new tools; PPG C2 contributors felt that the new tools would need promoting by a volunteer on site	Researchers should consider using to posters, large and small, co-designed with the PPIE group, to the requirements of each site (a, b outpatients, c1–c2); the toolkit guidance should be tailored to each site as follows: show both positive and negative feedback kiosk data; researchers should refresh the data to avoid posters looking static, to correspond with the new feedback reports for staff (sites a, b outpatients, c1–c2); increase the size of the poster from A4 to A0 where possible and use an arrow to advertise the tools volunteers on site, where possible, to promote the use of the tools (sites c2, b OPT)

## Abbreviations

UK: United Kingdom
PPIE: Patient and public involvement and engagement
PPGs: Patient and participation groups
FFT: Family and friends test
DEPEND: Developing and Enhancing Patient Experience and Narrative Data
FG: Focus group
CMHT: Community Mental Health Team
OPT: Outpatients
NIHR: National Institute of Health Research
