# Supplementary material for: Co-designing new tools for collecting, analysing and presenting patient experience data in NHS services: working in partnership with patients and carers
Source: Res Involv Engagem. 2021 Nov 27;7:85. doi: 10.1186/s40900-021-00329-3 (PMC8626979; doi:10.1186/s40900-021-00329-3)
Supplement: Supplementary file 2 — Additional file 2. Example trigger discussion slides used in the co-design process. [file 40900_2021_329_MOESM2_ESM.docx]

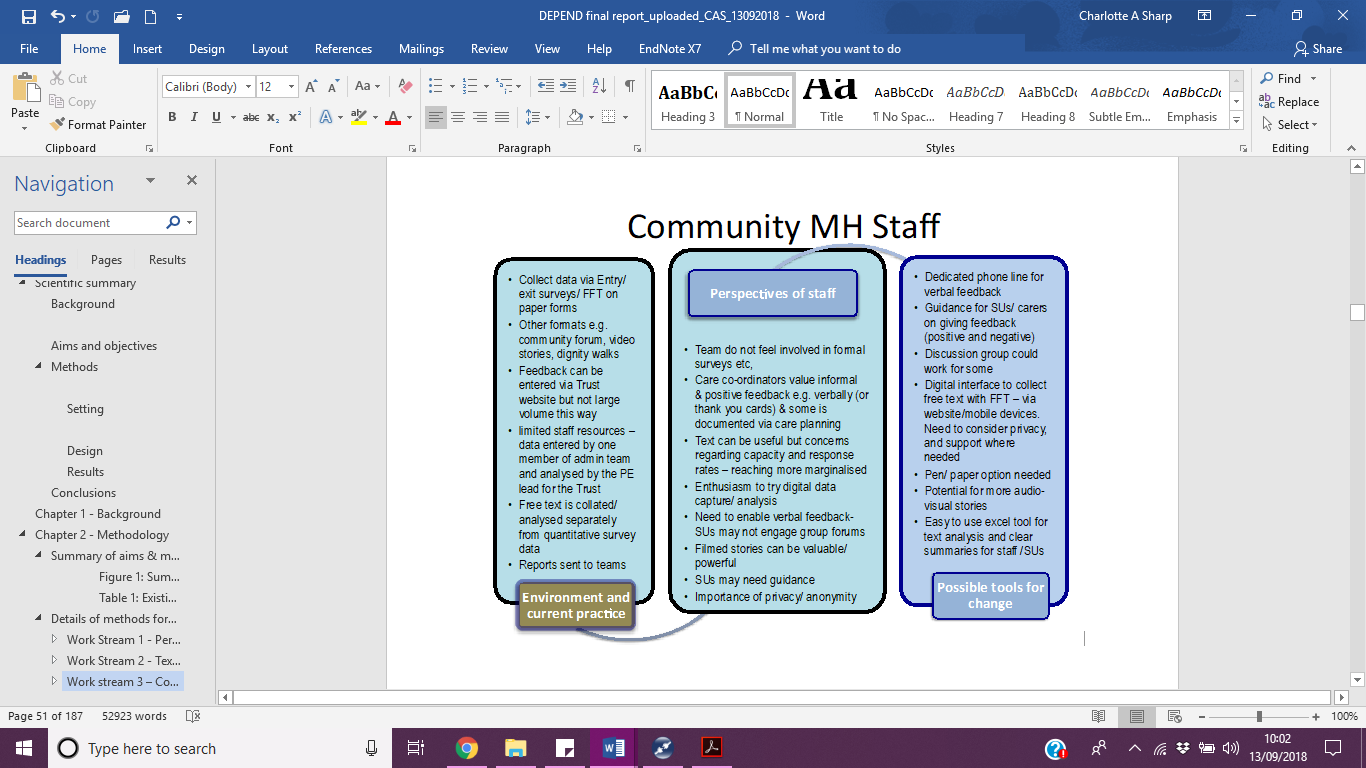


Additional file, Fig 1: Example trigger discussion slide from our co-design presentations to our PPIE group and research participants summarising links between current context, views of staff captured during work stream 1, and possible tools for change in Site B, to prompt co-design.


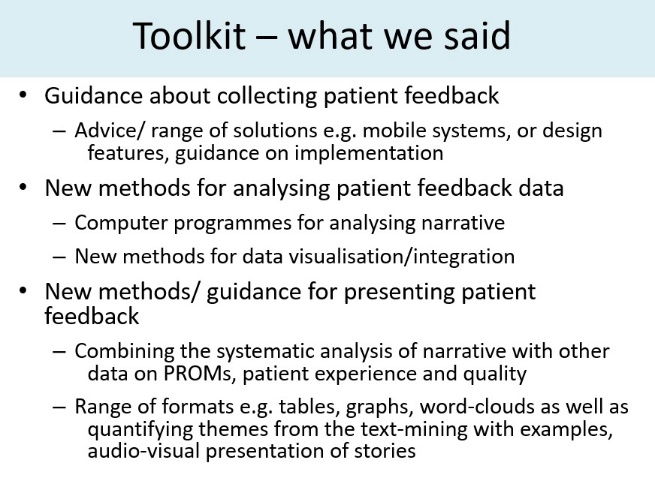


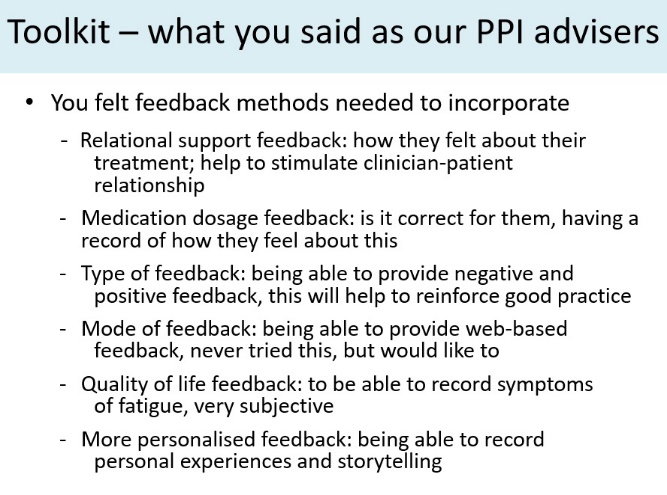

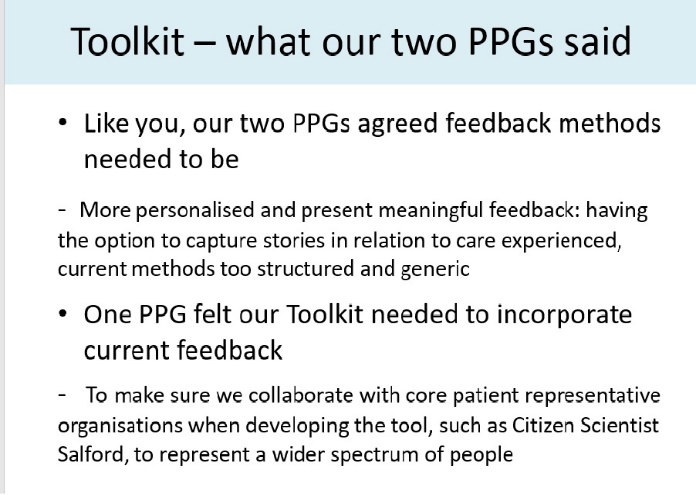


Additional file, Fig 2: Example ‘What we said, what you said’ trigger slides used during our PPIE in co-design discussions to enable shared experiences


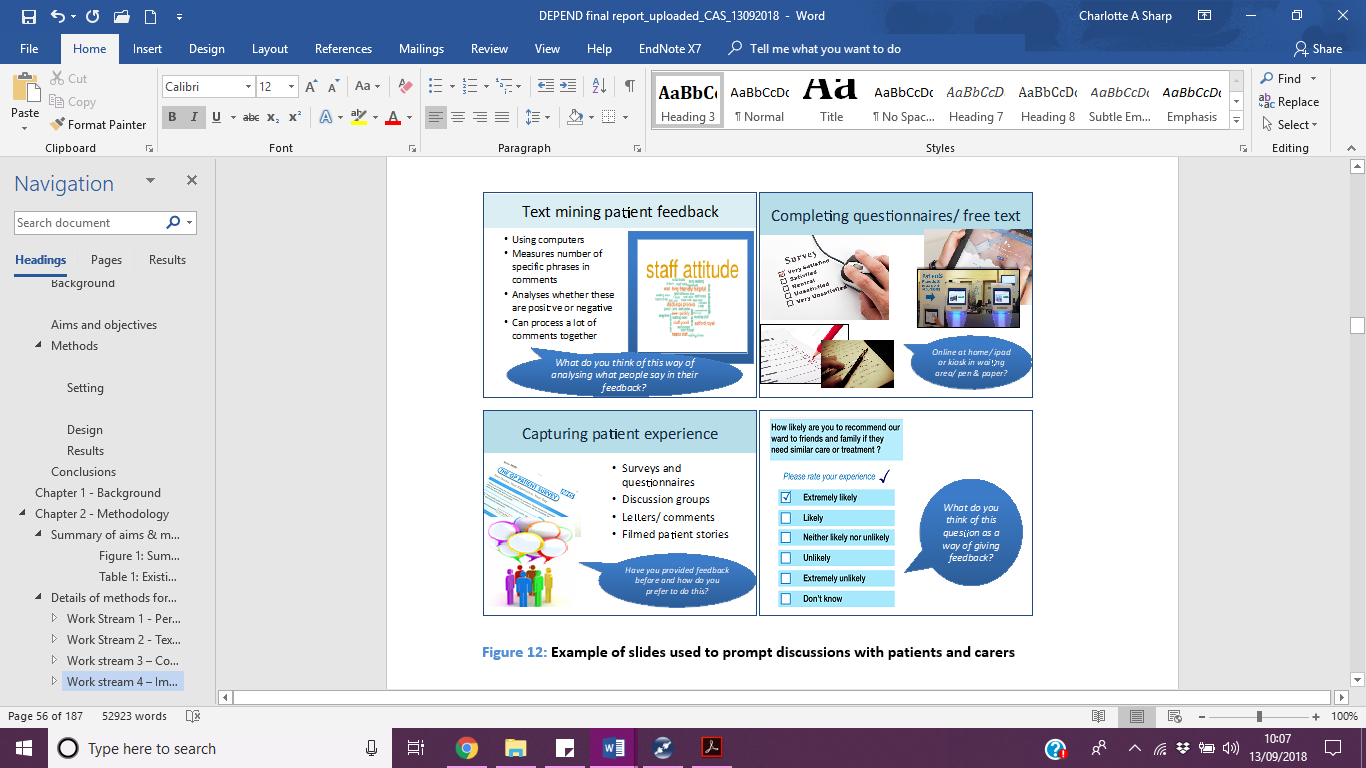


Additional file, Fig 3: Example slide prepared using insights from our PPIE group to prompt co-design discussions at interview and focus groups with patient and carer research participants.
