# Supplementary material for: Co-designing new tools for collecting, analysing and presenting patient experience data in NHS services: working in partnership with patients and carers
Source: Res Involv Engagem. 2021 Nov 27;7:85. doi: 10.1186/s40900-021-00329-3 (PMC8626979; doi:10.1186/s40900-021-00329-3)
Supplement: Supplementary file 3 — Additional file 3. The DEPEND study toolkit. [file 40900_2021_329_MOESM3_ESM.docx]

## DEPEND toolkit

- 1. A survey utilising the Friends and Family Test (FFT) with space for free text comments to be completed via digital kiosk within study sites, a website online, pen and paper version
  2. Guidance and information to support use of the new tools for:
     1. Staff
     2. patients and carers;
  3. New text mining programmes for analysing patient feedback data
     1. The open-source code for the text mining analysis is provided via the following website <http://gnteam.cs.manchester.ac.uk/depend/>
  4. New templates for reporting feedback
  5. A new process for eliciting and recording verbal feedback within community Site B services.

### A survey utilising the Friends and Family Test (FFT) with space for free text comments to be completed via digital kiosk within study sites, a website online, pen and paper version

#### Stills on Interface of kiosk in Sites A, B OPT, C1-C2

Trust Logo

Trust Logo

Site C1

Site C1

#### Online Survey

| **Site C1 Road Online Survey Draft** | | **Survey Created:** |
| --- | --- | --- |
|  |  | **Survey Changed:** |
| **Client:** DEPEND | | |
| **Survey:** Site C1 Medical Practice Survey ONLINE Version copy  **ID:** 14  **Link to DEMO version (use Internet Explorer)** [**https://secure.XXXXXX.com/Online/Survey/Preview/624A14**](https://secure.XXXXXX.com/Online/Survey/Preview/624A14) | | |
| **Start Message:** Please tell us what you think about our general practice service at Site C1 Medical Practice | **End Message:** Thank you for helping us to improve our general practice service at Site C1 Medical Practice. | |

**1. This is a short survey with two main questions. To keep your answers private, please do not enter any personal information such as names or contact details.**

*(Multiple Choice, select one only)*

- Press here to continue

**2. Are you a patient at Site C1 Medical Practice?**

*(Multiple Choice, select one only)*

- Yes Route to: Q4
- No

**3. Do you provide care for someone who uses the general practice service at Site C1 Medical Practice?**

*(Multiple Choice, select one only)*

- Yes
- No Route to: Q9

**4. 1. How likely are you to recommend this general practice service to your friends and family if they needed similar care or treatment?**

*(Multiple Choice, select one only)*

- Extremely unlikely
- Unlikely
- Neither likely nor unlikely
- Likely
- Extremely likely

**5. 2. Please tell us about your experience: (including good, bad or just ok)**

*(Open Ended)*

- Free Format Text

**6. Are you? (optional question)**

*(Multiple Choice, select one only)*

- Male Route to: Q8
- Female Route to: Q8
- Other Route to: Q7
- I prefer not to say Route to: Q8

**7. Please specify**

*(Open Ended)*

- Free Format Text

**8. What age are you? (optional question)**

*(Multiple Choice, select one only)*

- 0-15 years Route to: End
- 16 -24 years Route to: End
- 25-34 years Route to: End
- 35-44 years Route to: End
- 45-54 years Route to: End
- 55-64 years Route to: End
- 65-74 years Route to: End
- 75-84 years Route to: End
- 85 years Route to: End
- I prefer not to say Route to: End

**9. Thank you for your interest in giving feedback. At the moment this survey is only for people who use the general practice service here at Site C1 Medical Practice.**

*(Multiple Choice, select one only)*

- Press here to end the survey Route to: End

### Guidance and information to support use of the new tools: Site C1 poster

Site C1

Trust Logo

Site C1 URL

#### Kiosk Leaflets used in all sites to promote the use of the new tools

**
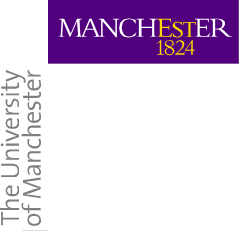

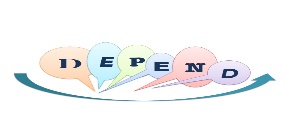
**

Trust Logo

- **The depend study is to improve the feedback system**
- **Tell us about your experience of our service at (Site C1)**
- **You can leave your feedback by the**
- **Machine (standing Kiosk) or**
- **Pen and paper or**
- **Online from home [http://bit.ly/XXXXXX]**
- **You can ask any staff for further queries**

**All those who take part will receive a High street voucher as a thank you for your time**
