# Supplementary material for: Co-designing new tools for collecting, analysing and presenting patient experience data in NHS services: working in partnership with patients and carers
Source: Res Involv Engagem. 2021 Nov 27;7:85. doi: 10.1186/s40900-021-00329-3 (PMC8626979; doi:10.1186/s40900-021-00329-3)
Supplement: Supplementary file 4 — Additional file 4. A new approach to collecting verbal feedback. [file 40900_2021_329_MOESM4_ESM.docx]

**A new approach to collecting**

**and documenting feedback from**

**service users for quality improvement**

**What is the DEPEND project and why is a new approach needed?**

The study is investigating the best ways to collect and use feedback from service users and carers to make it useful for quality improvement within specific staff teams. The Trust is working in collaboration with the University of Manchester for this project. Based on our initial stage of the research, we found that staff, service users and carers thought that survey questions are not always helpful. This is because the questions are very general, and many people will not respond, especially when unwell. Staff and service users talked about the value of face-to-face discussion about experiences of mental health services. This was viewed to be something that happens anyway during visits and conversations but is not always documented in detail or systematically.

**What is the new approach?**

The new approach suggested by the team is to collect and document feedback based on their visits systematically and formally within the ‘plan, outcome, action’ field of the electronic care record.

The team have also drafted some questions that might help to trigger this discussion routinely at the end of a visit. However, use of questions needs to be flexible and sensitive to individual circumstances and context of each visit.

Capturing the discussion formally as feedback will provide a more sensitive and inclusive process. This should also be more consistent with a recovery model of care. It might help to make feedback more meaningful and useful to individual staff, the team locally, and the wider Trust.

Having discussions about experience of service formalised as feedback and analysed in an aggregated way will also allow common issues to be identified and reflected upon at team meetings, and enable these to inform best practice.

**How should staff tell service users about the new approach?**

At home visits, staff should let service users and carers know that as a team you are testing out a new approach to the way you currently collect feedback and explain this. You may like to use this sheet as a guide, or you could say something like this to introduce the new approach…

*“As a team, we are starting a* ***new approach*** *to asking for feedback on how people experience our services. At each visit, I will ask you if you have any feedback that I can note down. This will enable us to ensure we can give the best possible service and support”*


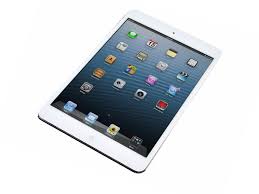

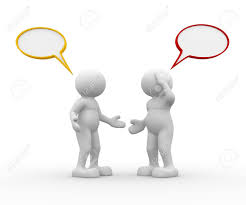


Staff need to ensure service users know that they are not under any pressure to give feedback in this way, and they can also give feedback using the usual anonymous surveys done within the Trust.

**Initiating a discussion about experience of service**

The team recognise that sometimes feedback is given naturally in discussions with service users, and sometimes people ask specifically about this at the end of a visit, but this varies according to circumstances. Sometimes specific questions can trigger a discussion to enable clients to give feedback. The team have drafted a few questions (see Table 1) as a guide to prompt such discussions.

**Table 1: Possible questions**

| **Example trigger questions** |
| --- |
| How are you feeling after today’s visit?  Can you say what has been helpful, or unhelpful?  Is there anything that you have found difficult?  What are your views about what we’ve talked about today?  Have we addressed all the issues needed today?  Have we covered all the questions that you’d like to ask?  Have your expectations been met today? [Possible prompts: timekeeping, communication]  Was everything useful [or ‘of value’] that we discussed today?  Is there anything you’d like to change [or, ‘look at in more detail’] as a result of today’s visit?  What shall we plan in terms of the next visit? |


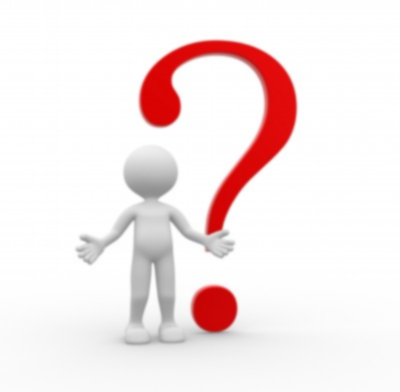
**Documenting the feedback?**

The care coordinator will record the discussion about their experiences of care at the end of the visit within the ‘plan, outcome and action’ field in the electronic care record. Please note down any discussions or comments that are relevant, and if you used a specific question. It is also helpful to record reasons why feedback is not documented; for example, if the circumstances made this difficult, or if the client did not want to give any feedback. This will help us to understand how useful this new process is.

**When will this new approach start?**

We will start the new approach of collecting and documenting feedback at the beginning of September 2017 and it will be tested over a period of approximately 6 months until the end of February 2018.

**How will the team and the Trust leads receive the analysis and presentation of feedback?**

The DEPEND team will summarise and produce a report of what feedback is given in this way each month. We will liaise with the team to consider how best to summarise information to make it most

useful for the team. We will also summarise issues raised by the team regarding the process itself, including any benefits and problems.

**What other methods are available for service users and carers to give their feedback?**

The Trust is also introducing a new programme of collecting feedback digitally using ipad devices in specific areas around the Trust. Service users can also give their feedback using an online version of the questionnaire, available here: [ xxxxxxxxxxxxxxxxxx ] or using the QR code here

However, we know it is unlikely that one method of collecting feedback will suit all clients. Service users and carers will still be able to complete the existing pen and paper questionnaires to give their feedback. The idea is that this new process of collecting verbal feedback being tested out by the community team will provide an additional way of enabling people to give feedback. Importantly, the new process should provide more useful information for the team to reflect on their specific practice, enabling ongoing high quality service provision.

**What support is available for the team?**

There is a DEPEND researcher, Nicola Small (Nic), who will support your team throughout this 6 months to encourage the new process of collecting and documenting feedback to evolve and improve and embed into everyday practice. Nic will be observing how this works in practice. Please contact Nic if you have any questions:


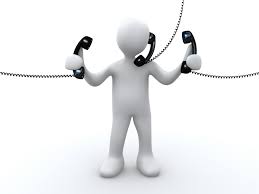


**DEPEND Project researcher:**

Nicola Small

Email: Nicola.small@manchester.ac.uk
